# Novel hepatotoxicity biomarkers of extracellular vesicle (EV)-associated miRNAs induced by CCl4

**DOI:** 10.1016/j.toxrep.2020.05.002

**Published:** 2020-05-29

**Authors:** Ryuichi Ono, Yusuke Yoshioka, Yusuke Furukawa, Mie Naruse, Makiko Kuwagata, Takahiro Ochiya, Satoshi Kitajima, Yoko Hirabayashi

**Affiliations:** a Division of Cellular and Molecular Toxicology, Center for Biological Safety and Research (CBSR), National Institute of Health Sciences (NIHS); b Division of Molecular and Cellular Medicine, Institute of Medical Science, Tokyo Medical University; c Central Animal Division, National Cancer Center Research Institute; d Division of Molecular and Cellular Medicine, National Cancer Center Research Institute; e Center for Biological Safety and Research (CBSR), National Institute of Health Sciences (NIHS)

**Keywords:** Exosome, Extracellular vesicle, EV, Hepatotoxicity, miRNA, Biomarker, Carbon tetrachloride (CCl4), miR-122, miR-192, miR423, miR-29c, ALT, AST

## Abstract

Recent findings have revealed that extracellular vesicles (EVs) are secreted from cells and circulate in the blood. EVs are classified as exosomes (40−100 nm), microvesicles (50−1,000 nm) or apoptotic bodies (500−2,000 nm). EVs contain mRNAs, microRNAs, and DNAs and have the ability to transfer them from cell to cell. Recently, especially in humans, the diagnostic accuracy of tumor cell type-specific EV-associated miRNAs as biomarkers has been found to be more than 90 %. In addition, microRNAs contained in EVs in blood are being identified as specific biomarkers of chemical-induced inflammation and organ damage.

Therefore, microRNAs contained in the EVs released into the blood from tissues and organs in response to adverse events such as exposure to chemical substances and drugs are expected to be useful as novel biomarkers for toxicity assessment. In this study, C57BL/6 J male mice orally dosed with carbon tetrachloride (CCl4) were used as a hepatotoxicity animal model. Here, we report that not only the known hepatotoxicity biomarkers miR-122 and miR-192 but also 42 novel EV-associated biomarkers were upregulated in mice dosed with CCl4. Some of these novel biomarkers may be expected to be able to use for better understanding the mechanism of toxicity. These results suggest that our newly developed protocol using EV-associated miRNAs as a biomarker would accelerate the rapid evaluation of toxicity caused by chemical substances and/or drugs.

## Highlights

• Establishment of a highly sensitive “next generation-type” toxicity test for chemical substances and drugs.
• Isolation of novel hepatotoxicity biomarkers of extracellular vesicle (EV)-associated miRNAs induced by CCl4.
• miR-122 and miR-192 are highly sensitive hepatotoxicity biomarkers equivalent to ALT and AST.

## Graphical abstract

**Figure:**
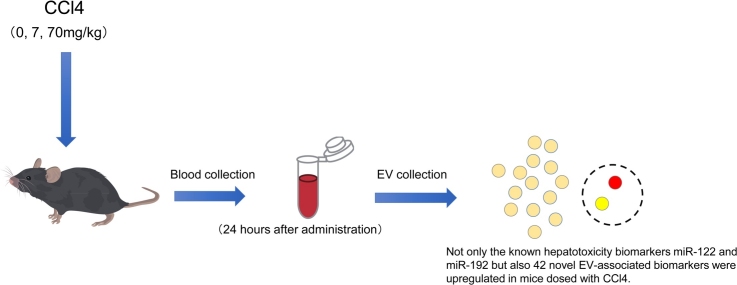

## 1 Introduction

Exosomes are a subset of extracellular vesicles (EVs), which are lipid bilayer vesicles secreted from cells that have a diameter of 40–100 nm [1]. Exosomes are endosomal in origin and contain proteins, lipids, DNA, mRNA, noncoding RNA, and small RNAs and are important mediators of cell-cell communication and horizontal gene transfer [2] [3] [4]. Exosomes were first discovered in 1983 and named “exosomes” in 1987 [5] [6]. To date, several types of EVs other than exosomes have been identified. They include microvesicles (50–1000 nm) and apoptotic bodies (500–2000 nm). They are distinguished by their size and mechanism of biogenesis [7]. In this study, we collected EVs, including exosomes, from mouse serum by differential ultracentrifugation; however, we could not exclude the possibility of contamination by microvesicles and apoptotic bodies. Therefore, we will use the term “EVs” as recommended by the International Society for Extracellular Vesicles, which includes vesicles present in the extracellular space (exosomes, microvesicles and apoptotic bodies) [8].

Recent reports demonstrate that EVs circulating in various fluids could be a diagnostic biomarker for various cancers [9] [10] [11] [12] [13]. EV-associated biomarkers are more sensitive and accurate than other widely used biomarkers, such as carcinoembryonic antigen (CEA) for adenocarcinoma and prostate-specific antigen (PSA) for prostate cancer. Furthermore, microRNAs contained in the EVs secreted from various cell types and human tissues have been identified as specific biomarkers of chemical-induced inflammation [14] [15] [16] [17] [18]. In addition, EV-associated miRNAs are well protected owing to the lipid bilayer membrane of EVs, even when EVs are purified from the circulating blood stream [19].

Therefore, we focused on EV-associated miRNA in blood as a biomarker to establish a highly sensitive "next generation-type" toxicity test for chemical substances and drugs. The aim of this study was to screen EV-associated miRNAs as novel biomarkers of hepatotoxicity by comprehensive RNA-seq analyses of EV-associated miRNAs in C57BL/6 J male mice orally dosed with carbon tetrachloride (CCl4). First, we established more accurate protocol for evaluating rodent EVs. Then, we screened and evaluated differentially expressed EV-associated miRNAs in response to the administration of hepatic toxicant, carbon tetrachloride (CCl4). We used the known hepatotoxicity biomarkers, miR-122 and miR-192, as positive controls for the screening [20] [21] [22] [17] [18] [23] [24].

## 2 Materials and methods

### 2.1 Animals

All animal studies were conducted in accordance with the guidelines of the animal care committee of the National Institute of Health Sciences (NIHS) (No. 664). C57BL6/J mice that were 12 weeks old were purchased from Charles River Laboratory Japan (Yokohama). The animals had access to a standard chow diet and water ad libitum and were housed in a pathogen-free barrier facility with a 12 L:12 D cycle.

### 2.2 Sample preparations

To produce the hepatotoxicity models, carbon tetrachloride (CCl4) (Fuji Film Co. Ltd, Tokyo, Japan) (0 (corn oil), 7, or 70 mg/kg body weight) was orally administered to C57BL/6 J mice. Twenty-four hours after the administration of CCl4, blood and liver sample collections were performed under isoflurane anesthesia. Whole blood was collected by cardiac puncture, kept undisturbed at room temperature for 30 min and centrifuged at 3000 x g for 10 min at 4 ℃, and the serum was carefully separated. The separated serum was stored at -80 ℃ until use.

### 2.3 Histology

Liver samples were fixed with 10 % neutral buffered formalin. After conventional processing, paraffin-embedded sections were stained with hematoxylin and eosin (H&E) and examined histopathologically under a light microscope.

### 2.4 Serum biochemistry analysis

The levels of aspartate aminotransferase (AST) and alanine aminotransferase (ALT) in each serum sample were determined using the automatic blood chemistry analyzer Dry-Chem NX 500 V (Fuji Film Co. Ltd, Tokyo, Japan).

### 2.5 EV analysis

The serum was centrifuged at 10,000 x g for 10 min to remove cellular debris and subsequently ultracentrifuged at 210,000 x g for 30 min (BECKMAN−COULTER Optima TLX). The pellet was resuspended in PBS and ultracentrifuged at 210,000 x g for 30 min again. The supernatant was carefully removed, and the EV pellets were resuspended in 30 μl PBS and stored at -80 ℃. Nanoparticle tracking analysis (NTA) of the EVs was performed with a Nanosight system (Nanosight) in EV diluted 1000-fold with PBS as previously described [25].

### 2.6 RNA extraction and mRNA-Seq library preparation

For EV-associated RNA, EVs were lysed with TRIzol, and EV-associated RNAs were extracted by a miRNeasy microelution kit (Qiagen) according to the manufacturer’s protocols. EV-associated RNA was evaluated by using a NanoDrop spectrophotometer (Thermo Fisher Scientific, Waltham, MA), an Agilent 2100 Bioanalyzer and RNA pico chips (Agilent Technologies, Palo Alto, CA).

cDNA was prepared from the EV-associated RNAs with a SMARTer smRNA-Seq Kit for Illumina (Clontech/TAKARA, Kyoto, Japan) according to the manufacturer’s protocol. Briefly, cDNA synthesis with the addition of both 5′ and 3′ adapters was followed by polyadenylation of EV-associated RNA (1 ng), first-strand synthesis and tailing with MMLV-derived PrimeScriptTM reverse transcriptase (RT), template switching with PrimeScript RT and the addition of full-length Illumina adaptors by PCR. The cDNA libraries were size-selected by using 3% agarose gel cassettes for the Blue Pippin System (Sage Science) with the parameters BP start = 148 and BP end = 185.

The quality and quantity of the size-selected cDNA libraries were checked with KAPA Library Quantification Kit Illumina® Platforms (Nippon Genetics, Japan) or Qubit dsDNA HS (High Sensitivity) Assay Kit in a Qubit® 2.0 Fluorometer (Life Technologies, CA, USA). 2.0 pM of the cDNA library for each sample was sequenced on an Illumina Nextseq500 platform, and 75-bp reads were generated (Illumina, USA).

### 2.7 RNA sequencing analysis

The raw data (raw reads) in the FASTQ format were first processed using the BCL2-FASTQ program (Illumina, USA). All the data analyses were performed on the Galaxy platform (https://usegalaxy.org). The FASTQ quality filter was performed using the Filter FASTQ program, and both the 5′ and 3′ adapters were trimmed using the Trim FASTQ program. These processed sequence reads were aligned to the reference genome (mm10) using the TopHat program to generate BAM files.

The BAM files were processed and normalized against the mouse miRNA database (miRBase: http://mirbase.org) by the Cufflinks and Cuffnorm programs to generate gene expression data for each miRNA. The RNA-Seq data were analyzed based on the fold changes and Hierarchical clustering between samples, which were calculated with a two-tailed Student’s *t*-test (P < 0.01) using the Subio Platform and Subio Basic Plug-in (v1.4; Subio Inc., Kagoshima, Japan).

### 2.8 qRT-PCR analysis for the validation of the RNA-Seq data

Real-time quantitative reverse transcription-PCR (qRT-PCR) was used to validate the RNA-Seq data. The cDNA libraries prepared for RNA-Seq were used to perform qRT-PCR. By using KAPA Library Quantification Kit Illumina® Platforms (Nippon Genetics, Japan), real-time absolute quantification of each cDNA libraries was performed. By using 5′ adapter primer and a reverse primer for each specific miRNA, real-time absolute quantification of miRNAs was performed. The primer sequences used in the qRT-PCR assay are as follows; The 5′ adapter primer: AATGATACGGCGACCACCGA, miR-122−5p-specific primer : CAAACACCATTGTCACACTCCA, miR-192−5p-specific primer : GGCTGTCAATTCATAGGTCAG. Real-time quantitative PCR analyses were performed with a ABI7500HT instrument (Applied Biosystems, Hercules, FL, USA); individual reactions were prepared with 0.2 pM of cDNA and Powerup SYBR Green Master Mix (Thermo Fisher Scientific) in a final volume of 20 μL. All reactions were performed in triplicate. Absolute quantification of miR-122a-5p and miR-192−5p was performed by using a standard curve method based on ten-fold serial dilution of synthesized oligo DNA miR-122−5p-Standard oligo: AATGATACGGCGACCACCGAGATCTACACTAGATCGCTCGTCGGCAGCGTCAGATGTGTATAAGAGACAGGGCTGGAGTGTGACAATGGTGTTTG, and miR-192−5p- Standard oligo:AATGATACGGCGACCACCGAGATCTACACTAGATCGCTCGTCGGCAGCGTCAGATGTGTATAAGAGACAGGGCCTGACCTATGAATTGACAGCC, raging from 5.0 × 10^6^ – 5.0 × 10° copies/PCR.

### 2.9 Statistical analysis

All data are presented as the means ± standard deviation (SD), and comparisons were performed by analysis of variance (ANOVA) (SAS). A probability of less than 0.01 was considered to be statistically significant.

## 3 Results

### 3.1 Isolation and characterization of EVs from mouse blood

**Fig. 1:**
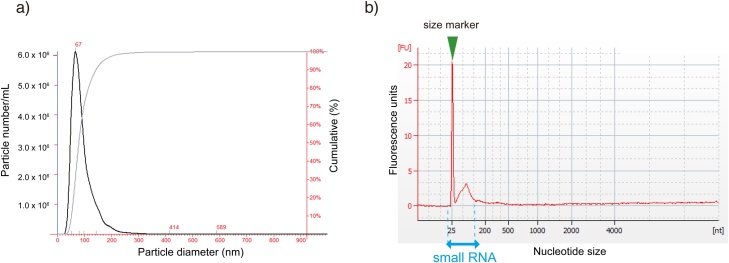
Characterization of EVs isolated from C57BL/6 J (12-week-old) male mouse serum. (a) Analysis of the particle size distribution of EVs from C57BL/6 J (12-week-old) male mouse serum by nanoparticle tracking analysis (NTA). Black line represents particle number and diameter. Gray line represents cumulative plot. (b) EV-associated small RNAs were analyzed by capillary electrophoresis. The profile is consistent with the presence of small RNAs but not long RNAs.

Whole blood was collected by cardiac puncture from C57BL/6 J male mice (12 weeks). EVs were purified by ultracentrifugation of the serum extracted from whole blood. Microparticle analysis of the EVs was performed with a Nanosight (Fig. 1a). The peak size of the EVs was 67 nm, which was comparable with that described in previous reports [26] [27]. For the analysis of EV RNAs, the total RNA extracted from the EVs was evaluated using an Agilent 2100 Bioanalyzer RNA pico kit (Fig. 1b). The peak size of the EV RNA was approximately 20–150 nt, and there was no obvious peak for the 18S & 28S ribosomal subunits, suggesting that these RNAs were derived from EVs rather than cellular RNAs.

In general, these small RNAs are believed to be tRNA, microRNA (miRNA), snoRNA, and small RNA fragments. miRNAs, in particular, have attracted attention as biomarkers, as there have been several studies on the use of miRNAs as diagnostic biomarkers for tumors with high accuracy [28].

### 3.2 Preparation of cDNAs to quantify EV-associated RNAs

Each miRNA was first transcribed as a large precursor, which was termed the primary miRNA (pri-miRNA), by RNA polymerase II [29] [30]. Pri-miRNAs are processed in the nucleus to yield 65-nt pre-miRNA hairpin intermediates by the nuclear RNase III enzyme Drosha and are further processed into mature miRNA in the cytoplasm by the second RNase III enzyme Dicer [31] [32] [33] [34] [35].

**Fig. 2:**
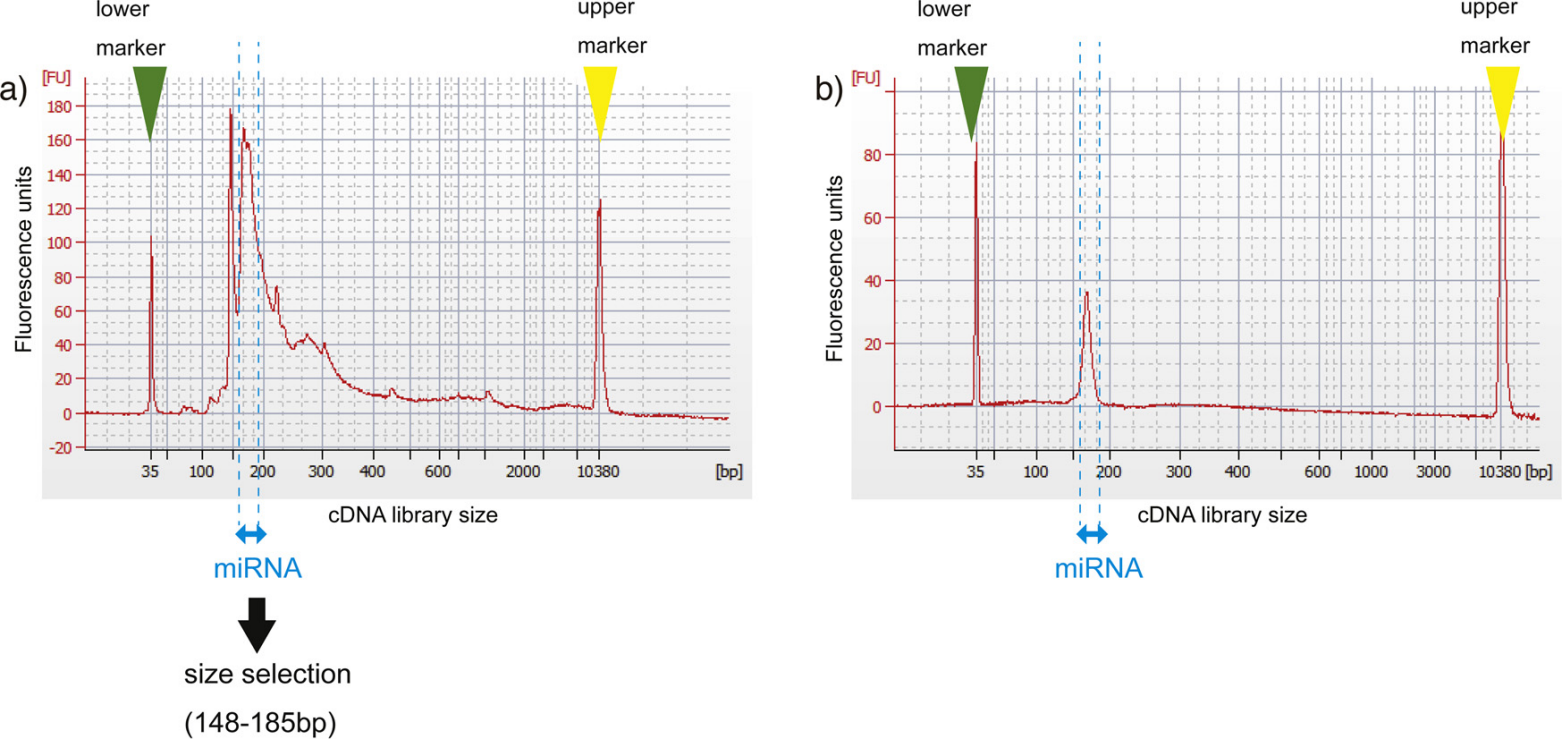
Characterization of the cDNA library of EV-associated miRNAs. (a) Electropherogram results for the cDNA library created from EV-associated small RNAs before size selection. The cDNA peak for EV-associated miRNAs with 5′ and 3′ adapter sequences corresponded to 176 bp. The region between 148 and 185 bp was selected for further characterization of EV-associated RNAs. (b) Electropherogram results for the cDNA library created from EV-associated small RNAs after size selection. The profile exhibits a narrow peak extending from 150 bp to 200 bp.

To quantify or sequence miRNAs, SMART (switching mechanism at the 5′ end of the RNA template) technology was used to generate cDNA libraries. Electropherogram results for cDNA library created from EV-associated small RNAs before size selection demonstrated that the region corresponding to the miRNA was a part of the whole library (Fig. 2a) [36].

Whether EV-associated RNAs were quantified by qRT-PCR or next generation sequencing (NGS), only the small RNA fractions of the cDNAs were further extracted by the size selector instrument to enrich the miRNA populations (Fig. 2b).

### 3.3 Carbon tetrachloride (CCl4) administration and histology

C57BL/6 J male mice were orally dosed with carbon tetrachloride (CCl4) as hepatic toxicant because there are numerous reports that CCl4 induces hepatotoxicity in many experimental animals [37]. In general, CCl4 is metabolized into the trichloromethyl radical by the drug-metabolizing enzyme P-450 in the liver. The trichloromethyl radical in hepatocytes reacts with oxygen to produce a trichloromethyl peroxy radical. These radicals cause lipid peroxidation and necrosis [38] [39]. Whole blood and liver samples were collected 24 h after the administration of CCl4 (0 mg/kg (vehicle control: corn oil), 7 mg/kg & 70 mg/kg) for the following research.

**Fig. 3:**
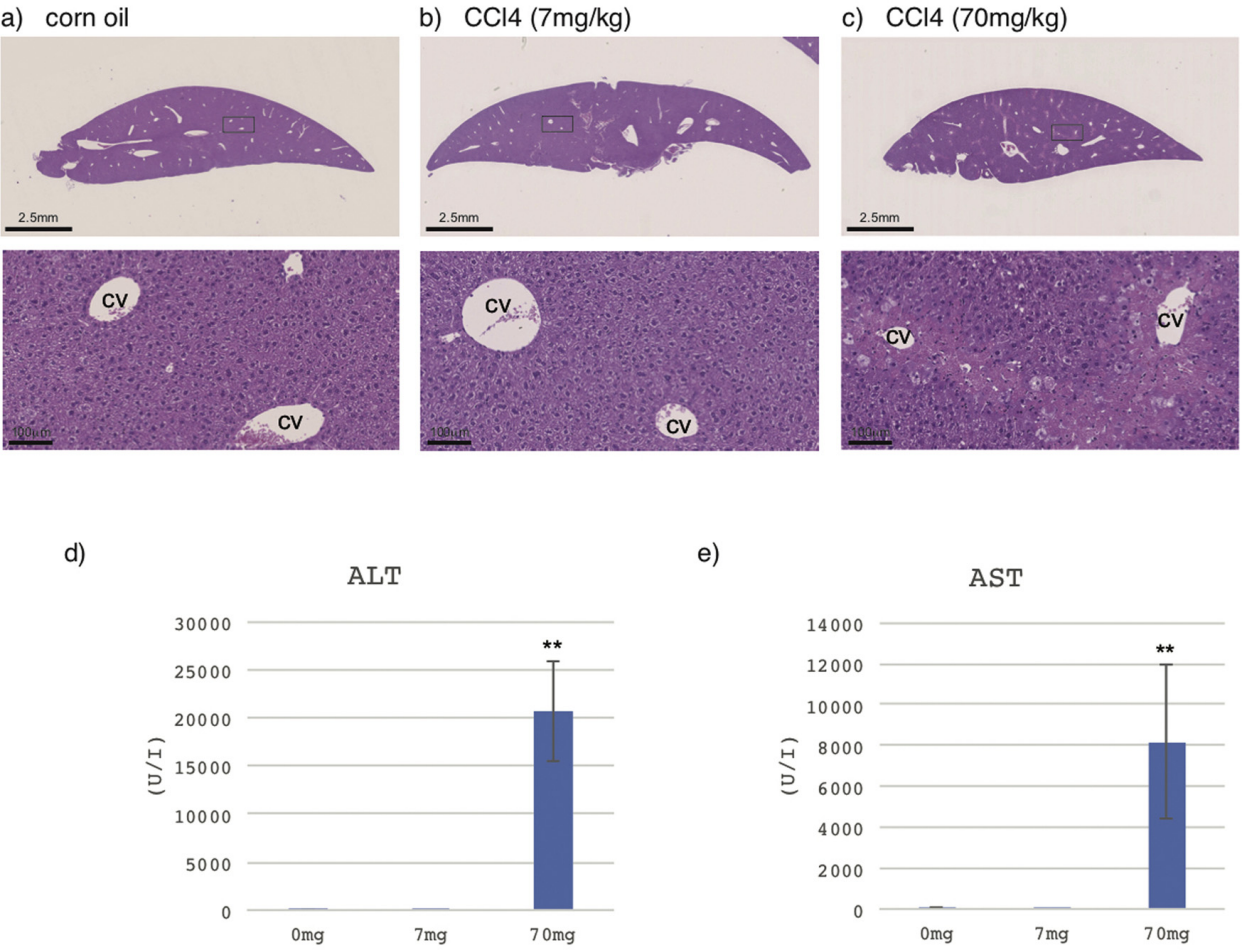
Representative H&E micrographs from liver tissues collected from mice treated with corn oil (control) (a), 7 mg/kg CCl4 (b), and 70 mg/kg CCl4 (c) by oral administration. The boxed areas in the upper photograph in a, b and c are magnified in the lower photographs in a, b, and c, respectively. Control section showing the normal histological structure of the central vein (cv) and surrounding hepatocytes (a). Twenty-four hours after 7 mg/kg CCl4 treatment, there were no histopathological changes compared with those in the control section (b). Twenty-four hours after 70 mg/kg CCl4 treatment, the hepatocytes around central veins (cv) were vacuolized and necrotic (c). Levels of AST (d) and ALT (e) in the serum of mice dosed with corn oil (0 mg/kg CCl4 control (N = 8)), 7 mg/kg CCl4 (N = 1), and 70 mg/kg CCl4 (N = 9) are shown. Data are expressed as mean ± SD, **P < 0.001, *P < 0.01 vs. control.

H&E staining showed CCl4 (70 mg/kg)-induced histopathological changes in the liver, with significant degeneration and necrosis of hepatocytes in the centrilobular region and the presence of perivenular inflammatory infiltrates 24 h after administration (Fig. 3c), while histological tissue sections of mice in the vehicle control group and the CCl4 (7 mg/kg) group showed normal histological morphology in the liver tissue samples (Fig. 3a, b).

### 3.4 Serum biochemistry analysis

The most commonly used diagnostic test for hepatotoxicity involves determining the activity of certain hepatocellular enzymes, including aspartate aminotransferase (AST or SGOT) and alanine aminotransferase (ALT or SGPT), in the blood [40]. AST is released into serum in proportion to cellular damage and is most elevated in the acute phase of cellular necrosis, whereas the release of ALT occurs early in liver damage and remains elevated for a relatively longer period.

AST (Fig. 3d) and ALT (Fig. 3e) levels were not increased by 7 mg/kg CCl4 but were increased by 70 mg/kg CCl4 compared to those in the corn oil group.

As a result, serum biochemistry analysis and histology of the results of CCl4 administration demonstrate that only 70 mg/kg CCl4 administration results in apparent hepatotoxicity.

### 3.5 Identification of differentially expressed miRNAs by RNA-seq

**Fig. 4:**
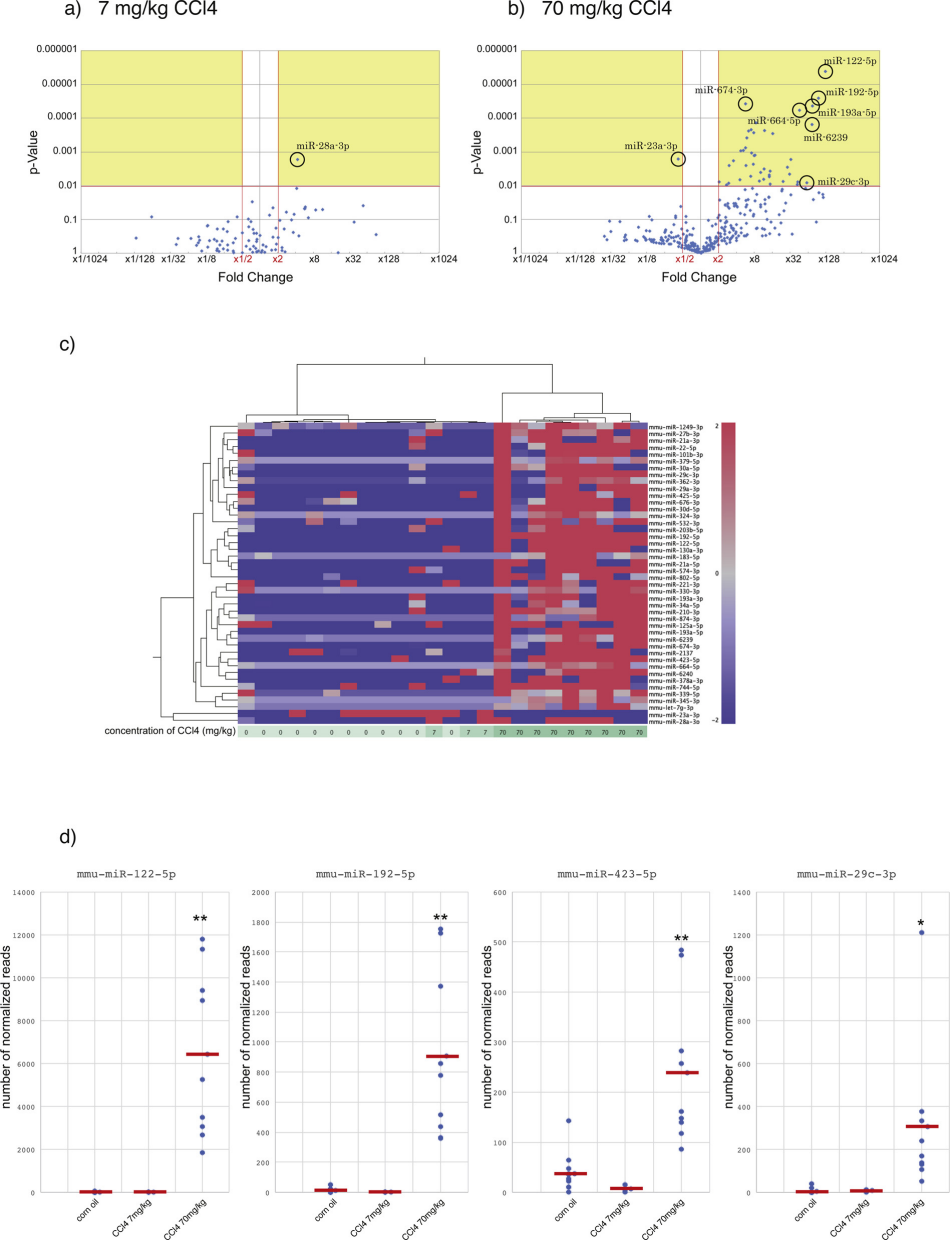
Differentially expressed EV-associated miRNAs. (a) Volcano plot of EV-associated miRNA RNA-seq data for corn oil oral administration (n = 12) and 7 mg/kg CCl4 oral administration (n = 3). (b) Volcano plot of EV-associated miRNA RNAseq data for corn oil oral administration (n = 12) and 70 mg/kg CCl4 oral administration (n = 9). The y axis values show the negative logarithm base 10 value of the P-value. The red horizontal line on the plot represents the α-level used for this analysis (0.01). The x axis is shown as the log2 difference in the estimated relative expression values. Vertical red lines represent the threshold of the log 2-fold change (equivalent to a 2-fold change). Thus, the dots in the beige-colored region correspond to miRNAs that show a significant (P < = 0.01) 2-fold or greater change in expression between the corn oil (control) and CCl4 (7 mg/kg (a), 70 mg/kg (b)) groups. (c) Heat map and hierarchical clustering of differentially expressed EV-associated miRNA data from the CCl4 (0, 7, 70 mg/kg) oral administration groups. 0 mg/kg (control) (n = 12), 7 mg/kg (n = 3) and 70 mg/kg (n = 9). The heatmap represents the results of the two-way hierarchical clustering of 44 differentially expressed EV-associated miRNAs and 24 samples. Each row represents a miRNA, and each column represents a sample. Upregulated miRNAs are shown in red, and downregulated miRNAs are shown in blue. Color densities indicating the levels of the fold changes are displayed. Individual normalized counts for four differentially expressed EV-associated miRNAs are shown (d). Red bar on the plots represents average of normalized counts. **P < 0.001, *P < 0.01 vs. control (For interpretation of the references to colour in this figure legend, the reader is referred to the web version of this article).

To identify differentially expressed EV-associated miRNAs, we performed RNA-Seq on size-selected, EV-associated small RNA libraries obtained from samples from mice treated with three dosages of CCl4, 0, 7, and 70 mg/kg. We then mapped the filtered clean reads to the reference mouse genome. We obtained an average of 68 million reads per sample that mapped to the reference genome. The library size-normalized counts for all samples were generated, and volcano plots of the differentially expressed EV-associated miRNAs are shown (Fig. 4). We identified 44 differentially expressed EV-associated miRNAs, including 43 upregulated genes and 1 downregulated gene, between the corn oil administration and 70 mg/kg CCl4 groups (Fig. 4b) (Table 1), while 1 differentially expressed upregulated EV-associated miRNA was identified between the corn oil administration and 7 mg/kg CCl4 groups (Fig. 4a) (p value ≤ 0.01 and an absolute value of the log2 ratio ≥ 1 were the threshold values). Hierarchical clustering was used to generate a heatmap of the top 44 differentially expressed EV-associated miRNAs, which showed a clear separation between the 70 mg/kg CCl4 administration group and the corn oil and 7 mg/kg CCl4 administration groups (Fig. 4c). Individual normalized counts were shown in four of 44 differentially expressed upregulated EV-associated miRNAs (Fig. 4d).

**Table 1:** List of miRNAs significantly up- and down-regulated induced by 70 mg/kg CCl4 oral administration ranked by P values. (N.A. indicates no expression in control samples).

miRNA	Fold change	P-value
mmu-miR-122−5p	124.3457982	4.10052E-06
mmu-miR-192−5p	95.40790089	2.52392E-05
mmu-miR-674−3p	5.706957637	3.73766E-05
mmu-miR-193a-5p	75.82387022	4.35667E-05
mmu-miR-192−3p	N.A.	4.80562E-05
mmu-miR-664−5p	45.71782006	5.85249E-05
mmu-miR-30d-5p	8.516113665	0.00013605
mmu-miR-6239	74.72199455	0.000155712
mmu-miR-1247−5p	N.A.	0.000174168
mmu-miR-28a-3p	7.083817232	0.000220674
mmu-miR-22−5p	11.76744086	0.000235333
mmu-miR-423−5p	6.388818576	0.000240415
mmu-miR-187−3p	N.A.	0.000252835
mmu-miR-130a-3p	7.862186383	0.000272001
mmu-miR-193a-3p	16.22203082	0.000282773
mmu-miR-210−3p	5.932110798	0.000387975
mmu-miR-1249−3p	5.668511989	0.000755986
mmu-miR-532−3p	5.228119523	0.000912155
mmu-miR-425−5p	4.768893321	0.000917652
mmu-miR-339−5p	7.229527922	0.00108171
mmu-miR-574−3p	8.261805959	0.001235294
mmu-miR-6240	7.923153144	0.001268117
mmu-miR-23a-3p	0.420764258	0.001636375
mmu-miR-874−3p	33.09392448	0.001684601
mmu-miR-21a-5p	3.546441934	0.001833359
mmu-miR-324−3p	7.69715977	0.0023448
mmu-miR-744−5p	3.003918983	0.002546474
mmu-miR-676−3p	5.767312941	0.002546729
mmu-miR-345−3p	28.52191857	0.002851029
mmu-miR-376b-3p	N.A.	0.003074675
mmu-miR-203b-5p	11.61612346	0.003215505
mmu-miR-21a-3p	14.70577286	0.003736011
mmu-miR-101b-3p	10.14888491	0.004098845
mmu-miR-183−5p	31.16821919	0.004545263
mmu-miR-455−5p	N.A.	0.004689289
mmu-miR-29a-3p	14.18293213	0.004851941
mmu-miR-330−3p	6.900663431	0.005282091
mmu-miR-362−3p	14.18493622	0.005383571
mmu-miR-378a-3p	11.52840894	0.006486874
mmu-miR-27b-3p	4.550345431	0.006635769
mmu-miR-30a-5p	9.760313913	0.007347957
mmu-miR-34a-5p	24.45306825	0.007460881
mmu-miR-125a-5p	2.042752023	0.007747797
mmu-miR-802−5p	42.76078307	0.00781044
mmu-miR-1843a-5p	N.A.	0.008113383
mmu-miR-29c-3p	61.4392178	0.008149335
mmu-miR-2137	2.958524964	0.008523622
mmu-miR-379−5p	14.77543169	0.009083035

### 3.6 qRT-PCR analysis for the validation of RNA-Seq data

**Fig. 5:**
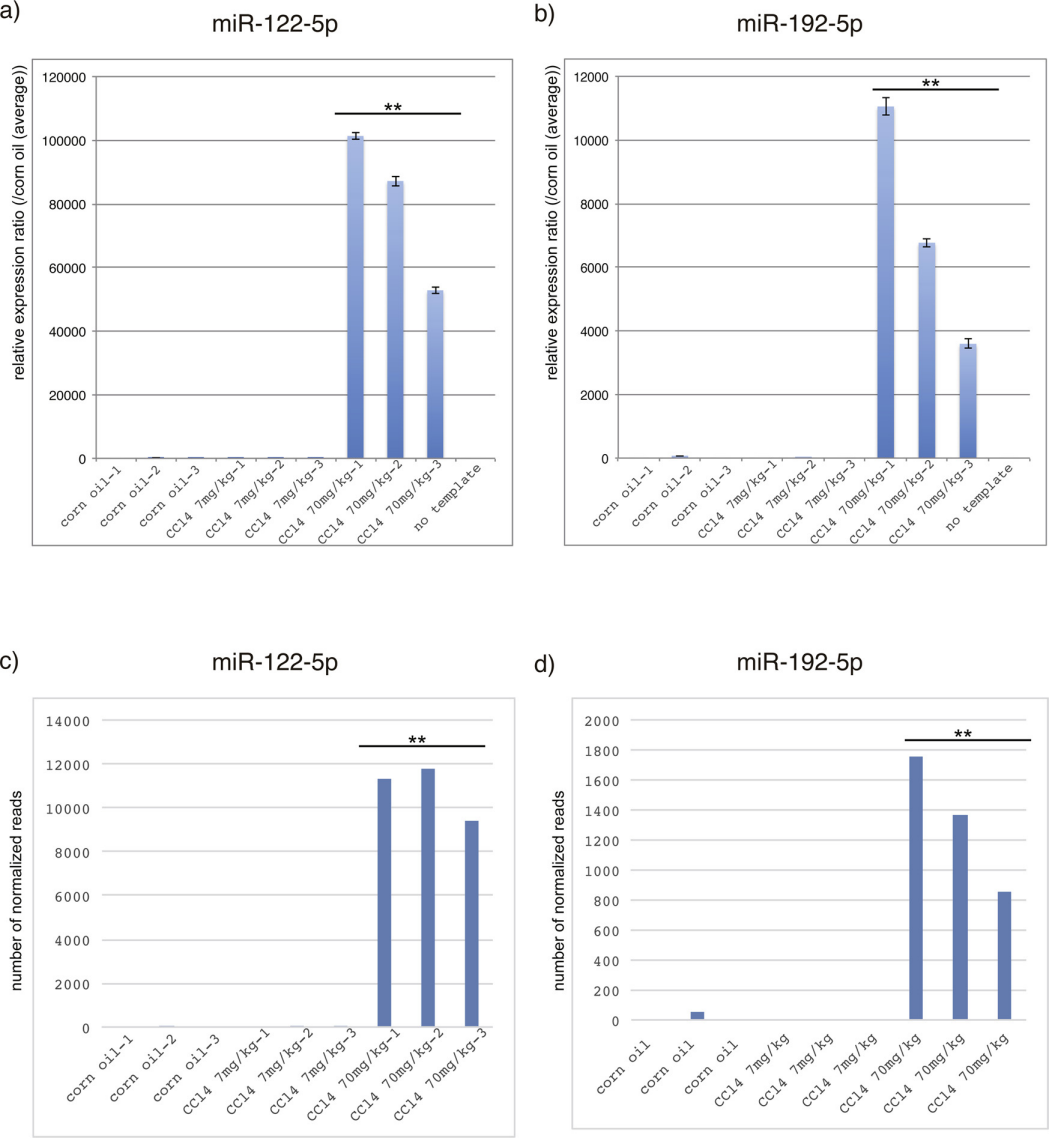
qRT-PCR analysis for the validation of RNA-Seq data. Increased numbers of EV-associated miR-122 and miR-192 were found to result from 70 mg/kg CCl4-induced liver injury by qRT-PCR. Absolute quantification of EV-associated miR-122 (a) and miR-192 (b) after 0, 7, and 70 mg/kg CCl4 administration was determined by using the standard curve method (n = 3 for each dose). qRT-PCR of miR-122 using corn oil-1, 3, CCl4 7 mg/kg-1 cDNA library templates and miR-192 using corn oil-1, 3, CCl4 7 mg/kg-1, 2, 3 cDNA library templates were below the detection limit (5 copies/PCR) and were given a value of 5 copies/PCR. The expression levels of EV-associated miR-122 (c) and miR-192 (d) calculated from the miRNA-seq data are represented. The cDNA library templates used for qRT-PCR and miRNA-seq were the same. **P < 0.001, *P < 0.01 vs. control.

To validate the RNA-Seq data, the known hepatotoxicity biomarkers, miR-122 and miR-192, were quantified in the same cDNA library as that used for RNA-seq by quantitative RT-PCR [20] [21] [22] [17] [18] [23] [24]. Levels of EV-associated miRNA-122 (Fig. 5a) and miRNA-192 (Fig. 5b) increased more than 1000-fold and 100-fold, respectively, due to the administration of 70 mg/kg CCl4 but did not respond to the administration of 7 mg/kg CCl4. These results are comparable to those of the NGS analyses (Fig. 5c, d), suggesting that the biomarkers identified by NGS are indicative of hepatotoxicity.

### 3.7 Discussion and conclusions

Liquid biopsy in medical examination is known as a very powerful tool. In fact, AST and ALT are known to be very good biomarkers of liver damage; however, it is difficult to distinguish the cause of liver damage or status of the liver based on only elevated levels of AST and ALT. And, further examination is needed for detailed lesion assessment in some cases. Recently, using EV-associated miRNAs or circulating free miRNAs in serum as biomarkers has made it possible to diagnose 13 types of cancer with an accuracy of 90 % or more [41] [42] [43] [44] [45] [46] [47] [48]. Therefore, the development of next-generation toxicity tests using miRNA as a biomarker is expected.

In this study, we succeeded in isolating 44 miRNAs containing the known liver damage biomarkers miR-122 and miR-192. Using those novel biomarkers, it may be possible to elucidate the mechanism of hepatotoxicity by analyzing the behaviors of biomarkers when hepatotoxicity is caused by administration of drugs or chemical substances other than carbon tetrachloride. Since EVs in blood are secreted by a wide variety of cells, it is unclear whether the EV-associated miRNAs identified as novel biomarkers are secreted only by the liver. And effort to evaluate the origin of EVs should be necessary, however, to understand the systemic toxicity, no matter where EVs come from, whole biomarker analysis may be important. Because in addition to hepatotoxicity, it is also possible to comprehensively detect systemic toxicity by proceeding with analysis of EV-associated miRNAs as biomarkers by targeting various organs such as the kidney, lung and heart.

Although this study analyzed the effects of carbon tetrachloride administrations at single time point, 24 h, clarifying the time courses of biomarkers in response to repeated administration of carbon tetrachloride may be applicable to shortening chronic toxicity tests and long-term carcinogenicity tests.

To date, circulating free miRNAs in serum have also been used in addition to EV-associated miRNAs as biomarkers of carcinogenesis. EV-associated miRNA is contained in serum RNA, but it is also known that some miRNAs have different expression profiles between EV-associated miRNA and circulating free miRNA in serum [49] [50]. Therefore, there are miRNAs that are actively incorporated into EVs. Considering these facts, miRNAs that are actively incorporated into EVs may be captured by other organs or cells as part of their function. It may be important to elucidate the function of EV-associated miRNAs induced by hepatotoxicity *in vivo.*

Finally, by quantification of EV-associated miR-122 and miR-192 using qRT-PCR and next-generation sequencing of the same serum sample, it was revealed that the miRNA expression profiles obtained from miRNA sequencing were almost identical to those obtained from qRT-PCR. Therefore, once useful biomarkers have been identified, systemic toxicity can be evaluated simply by performing qRT-PCR of each biomarker.

## Author contributions

R.O., T.O., S.K., and Y.H. conceived the study. R.O., Y.Y., and Y.F. participated in the experimental design and performed analyses. M.K. participated in histological analysis. R.O. and M.N. analyzed NGS data. R.O. wrote the manuscript. All authors read and approved the final manuscript.

## Funding

This work was supported in part by Research on the Regulatory Science of Pharmaceuticals and Medical Devices (20mk0101163j0202), Research on the Development of New Drugs (20ak0101093j003) from the Japan Agency for Medical Research and Development, the Health Sciences Research Grants from the Ministry of Health, Labor, and Welfare, Japan, (H30-KAGAKU-IPPAN-002), and JSPS KAKENHI (18K19315) to R.O. ; Grant-in-Aid from the Research Program on Hepatitis from Japan Agency for Medical Research and Development (AMED: 16fk0310512h0005, 17fk0310101h0001 and 18fk0310101h0002) and Grants-in -Aid from a Project for Cancer research and Therapeutic Evolution (P-CREATE) to T. O. ; JSPS KAKENHI (18K07053) to Y. H.

## Declaration of Competing Interest

The authors declare no competing interests.
